# Additive Manufacturing and Mechanical Performance of Trifurcated Steel Joints for Architecturally Exposed Steel Structures

**DOI:** 10.3390/ma13081901

**Published:** 2020-04-17

**Authors:** Pengfei He, Wenfeng Du, Longxuan Wang, Ravi Kiran, Mijia Yang

**Affiliations:** 1 Institute of Steel and Spatial Structures, College of Civil Engineering & Architecture, Henan University, Kaifeng 475004, China; 15537898976@163.com (P.H.); wlongxuan@126.com (L.W.); 2 Department of Civil and Environmental Engineering, North Dakota State University, Fargo, ND 58108-6050, USA

**Keywords:** trifurcated joint, additive manufacturing, AM process parameters, mechanical testing of AM parts, finite element analysis of AM parts

## Abstract

Additive Manufacturing (AM) technology has unique advantages in producing complex joints in architecturally exposed steel structures. This article focuses on the process of manufacturing and investigating the mechanical properties of a reduced scale model of a trifurcated joint using Selective Laser Melting (SLM) method and mechanical tests, respectively. The orthogonal test method was used to optimize the main AM process parameters. Then the trifurcated steel joint was printed using the optimal process parameters and treated by solid solution and aging treatment. To investigate the mechanical performance of the printed joint, an axial compression test and complimentary finite element analyses were carried out. Failure processes and failure mechanisms of the trifurcated steel joint were discussed in detail. The research results show that the preferred process parameters for printing 316L stainless steel powder are: scanning power 150 W, scanning speed 700 mm/s, and scanning pitch 0.09 mm. Using these AM parameters, trifurcated steel joints with good surface quality, geometrical accuracy and tensile strength are obtained after heat treatment. Our mechanical tests and Finite element analyses results indicate that the failure mechanism in the AM trifurcated joint are similar to those of cast steel joints. Based on these results, we conclude that the AM technology serves as a promising new way for the fabrication of joints with complex geometries.

## 1. Introduction

**Figure 1:**
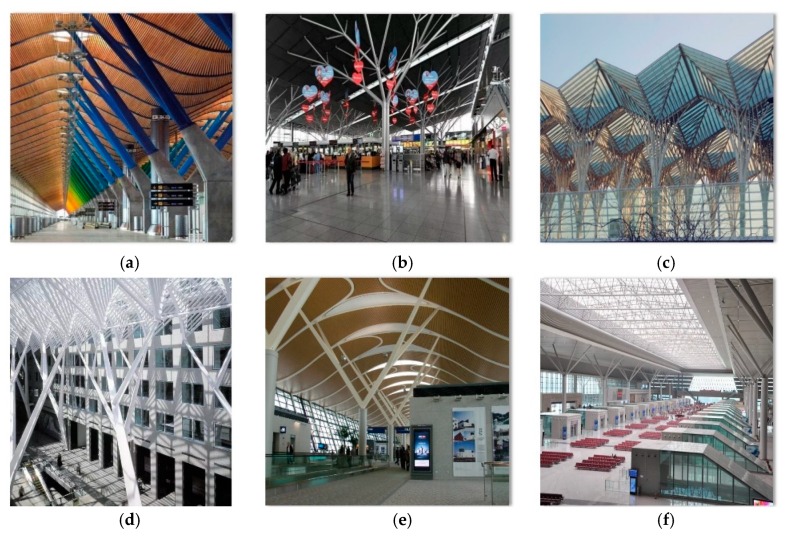
(**a**) New Delhi Airport Terminal T4; (**b**) Stuttgart Airport Terminal; (**c**) Lisbon Orient Station; (**d**) Allen Lambert Promenade; (**e**) Shanghai Pudong Airport T2 Terminal; (**f**) Xi’an North High-Speed Railway Station. (Images by Wen-Feng DU).

The cast steel joint is a one-piece connection and is generally used in connections involving complex structural members with no weld access available or when too many weld passes are required in the connection region. The use of cast steel joints eliminates the need for complicated welding activity in the joint area. This unique advantage resulted in a wide use of cast steel joints in architecturally exposed steel structures in the recent years. Especially in the past 10 years, nearly 50% of large-scale steel structural steel projects have adopted cast steel joints in the place of welded connections (see Figure 1) [1,2].

However, the current research on cast steel bifurcation joints is insufficient. Especially, there has been no significant progress for many years in the manufacturing process of steel bifurcation joints. The traditional casting process of steel joints leads to a lower geometrical precision, longer manufacturing periods, high energy consumption and less architectural freedom [3,4,5]. Moreover, cast steel joints have to be mass produced in order to be affordable and hence many numbers of different joints are casted at once to cut the costs limiting the architectural freedom. Technologies like Additive manufacturing (AM) are necessary in the place of traditional casting process to fabricate many types of joints in a single project without cost escalation.

AM technology is rapidly revolutionizing the manufacturing industry. AM is also widely used in the aerospace, mechanical, and biomedicine engineering, and has also received considerable attention in the field of civil infrastructure [6]. AM technology leverages the versatility of digitalized models and facilitates personalization and customization of components and is seen as a catalyst to the third industrial revolution [7]. Compared to traditional manufacturing methods, AM technology offers greater design flexibility, efficient material usage, and energy savings [8,9,10,11]. In the context of civil infrastructure, AM can provide exceptional architectural freedom for building aesthetically appealing architecturally exposed steel structures [12,13].

There have been some attempts in the past to create complex joints using AM technology. For example, Zhao Yang et al [14] employed topology optimization to design the joints in cable-strut structures and used AM to manufacture the optimized joints with complex geometry. Strauss H [15,16] used AM technology to fabricate the Nematox Façade joints. This new joint enables topology-optimized shapes, allowing the structure to achieve a free geometry. Arup [17] redesigned the joints of the overall tension structure and chose AM technology to make the molding. From the results in these few publications so far, it is feasible to manufacture joints by using AM technology, and has some outstanding advantages. However, there is currently a lack of research on joint performance made employing AM. The 3D printed material has surface unevenness and anisotropy, the phase composition of the metal is different from the traditional casting process due to the rapid solidification during AM process. These differences make it very meaningful to study whether the performance of joints manufactured by AM technology can meet the various performance requirements of practical engineering applications.

In this article, the mechanical performance of trifurcated joints fabricated employing AM technology was studied. Firstly, an orthogonal test method is used to select the manufacturing process parameters that influence the quality of fabricated joint. Subsequently, a typical tree-like three-forked trifurcation joint is fabricated by employing the previously determined process parameters. The mechanical performance of the fabricated three-forked bifurcation joint is demonstrated by conducting a monotonic loading test. Finally, the damage process and ultimate failure of the three-fork joint is analyzed by combining experimental results with numerical simulations.

## 2. Additive Manufacturing of Bifurcated Steel Joints

### 2.1. Metal Additive Manufacturing

According to the ISO/ASTM 52900:2015 [18], AM is a process of joining materials to make parts from 3D model data, usually layer upon layer, as opposed to subtractive manufacturing and formative manufacturing methodologies [19,20,21]. AM involves fusion of similar materials or adhesion of dissimilar materials and can be broadly classified in single-step AM process and multi-step AM processes. Single-step AM processes do not require a post-processing step like sintering and / or infiltration. Most of the metallic ceramic and composite parts require a post-processing step.

**Figure 2:**
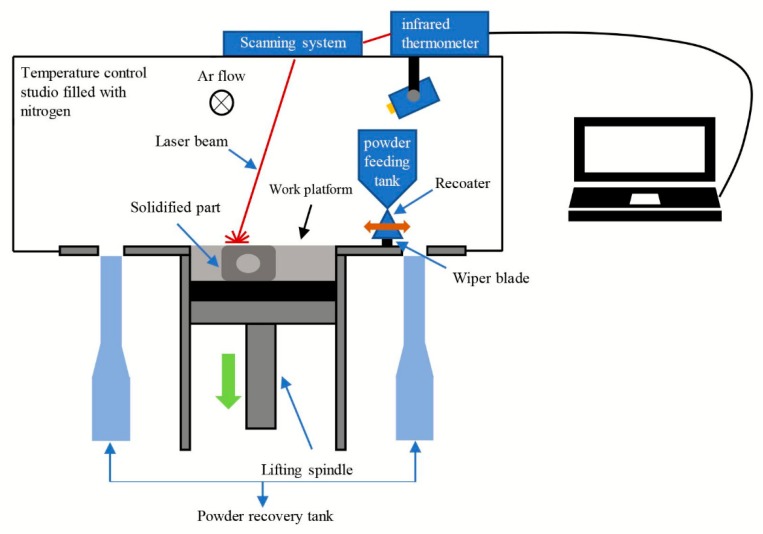
SLM technology molding schematic.

Selective laser melting (SLM), direct metal laser sintering (DMLS), selective laser sintering (SLS), laser engineered net shaping (LENS), and electron beam selective melting (EBSM) are some of the commercially available metal AM methods [22,23,24]. Among these methods, SLM is a relatively mature rapid prototyping technology. SLM can be used to obtain complex shaped functional components with bulk densities close to 100%. Moreover, high geometrical precision (0.05–0.3 mm) and low surface roughness (Ra 30–50 µm) are possible when SLM is used [25]. The printing equipment used in this study is HBD-200 metal 3D printer of Guangdong Han-bang Laser Technology Co., Ltd. (Guangzhou, China). This machine uses a high-energy laser beam to melt a bed of metal powder building the component layer by layer according to the section-by-section input from the three-dimensional metal solid part model. The entire process is carried out in a closed atmosphere filled with an inert gas to prevent the metal powder from reacting with the air at high temperatures generated by the laser, as shown in Figure 2. The maximum laser power of this machine is 200 W, the print layer thickness is 10–40 μm, the print line width is 40–80 μm, the line scanning speed is ≤10,000 mm/s, and the user can customize the process parameters.

There are three main causes of fatigue cracks in SLM moldings:

(1) Surface/ below-surface defects such as micro-voids in parts can cause stress concentrations and hence induce fatigue cracks.
(2) The anisotropy of additive manufacturing parts can also have a significant impact on fatigue performance.
(3) The high temperature gradient generated during the rapid cooling of the molten pool leads to the generation of tensile residual stress which in conjunction with micro-voids can accelerate the development of fatigue cracks.

However, the use of optimized printing process parameters and appropriate heat treatment can significantly reduce the defects, anisotropy and residual stresses, which can improve the fatigue resistance of SLM molded parts [26]. Therefore, it is very important to study the optimization of process parameters.

### 2.2. Material Selection

There are many kinds of commercially available spherical metal powders used for AM of metals, such as stainless steel, bronze-based metal, cobalt-chromium series, tool steel, titanium series, nickel alloy, aluminum alloy, and other metal powders. For structural steel applications, 316L stainless steel metal powder is often preferred. 316L has good gloss improving the appearance of the finished structural component, excellent corrosion resistance due to the addition of chromium which allows the formation of a nanoscale passive layer, and excellent high temperature mechanical strength which is a desirable quality in architecturally exposed steel structures. From an economic standpoint, the price of stainless steel 316L powder material is relatively lower compared to other powder materials making it desirable for the fabrication of building components. For the above reasons, 316L stainless steel metal powder is used in the AM of joints. 

Experiments were performed using aerosolized 316L stainless steel powder produced by Guangzhou Han-bang Laser Technology Co., Ltd. The chemical composition of stainless steel 316L powder material is: Mo (2.25–3%); Fe (balance); Cr (17–19%); Ni (13–15%); P (≤0.025%); S (≤0.010%); Cu (≤0.5%); C (≤0.03%); Si (≤0.1%); N (≤0.1%); and Mn (≤2%). The particle size distribution interval is 15–45 μm.

### 2.3. Selection of SLM Fabrication Process Parameters

In order to obtain high-density, it is necessary to optimize the process parameters of the SLM, such as scanning strategy, laser power, laser scanning rate, scanning pitch, and layer thickness. The ideal process parameters are not the same for different metal powders, and changing one process parameter will also influence the other process parameters. Some experiments are conducted to study the influence of laser power, scanning speed and scanning pitch which are the most important processing parameters with the aim of finding the ideal set of parameters. To this end, Taguchi analysis method was used to obtain more comprehensive information with fewer number of experiments [27]. 

**Figure 3:**
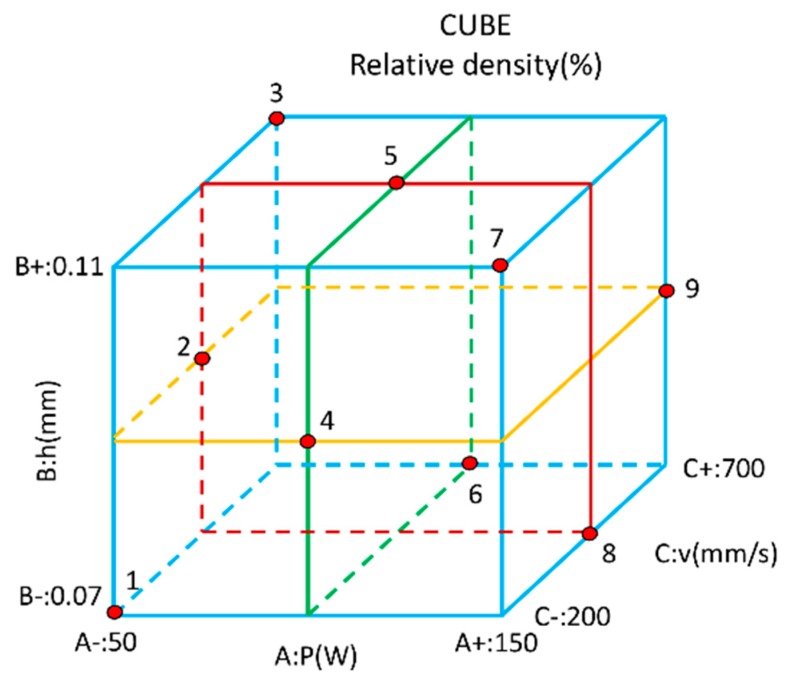
Schematic diagram of orthogonal test design.

The Taguchi analysis method uses orthogonal tables of experimental design theory to study a large number of variables in small experiments. Three levels of the three chosen processing parameters are used in the tests. As depicted in the cube in Figure 3, each level represents a plane, and there are 9 planes for all the 3 processing parameters. The cube has 27 sets of test points. The parameter factors and design levels of the orthogonal test are shown in Table 1, and the printed layer thickness is selected to be 30 µm [28]. The size of the sample prepared by the SLM process was about 10 × 10 × 9 mm. The relative density of the sample was measured by Archimedes principle. As we aim to obtain the parameters that yield maximum relative density, we chose the "Large characteristic" standard in Taguchi analysis method to calculate the Signal to Noise Ratio (SNR). The SNR formula is shown in Equation (1), where y is the result of the relative density test and n is the number of experiments. The calculation results are shown in Table 2.

(1) $$SNR=-10log_{10}\left(\frac{1}{n}\overset{n}{\underset{i=1}{\sum }}\frac{1}{y^{2}}\right)\;\;\;\;\;\;$$

It can be seen from Table 2 that the signal-to-noise ratio of the test no. 9 is the largest, so the no. 9 test scheme is the optimal set of parameters, and the corresponding optimal process parameters are: *P* = 150 W, *v* = 700 mm/s, *h* = 0.09 mm. 

Orthogonal tests often use the analysis of variance method to evaluate the error, and the results are summarized in Table 3.

Where *T* is the sum of *SNR*; ($T_{1},T_{2},T_{3})$ are the sum of *SNR* of each level in each column, (${\bar{T}}_{1},\;{\bar{T}}_{2},\;{\bar{T}}_{3}$) are their average values; *R* is the range of each column; $S_{T}$ is the synthesis of the sum of squared fluctuations of *SNR*; *S* is the synthesis of the sum of squared fluctuations of *SNR* in each column.

*SNR* analysis of variance:

Calculate the degrees of freedom between the factors in Equation (2)

(2) $$f_{A}=p-1=3-1=2$$

where, *r* is the number of factors, *p* is the number of levels, $f_{A}$ is the degree of freedom between factors.

Calculate the degrees of freedom of the error in Equation (3)

(3) $$f_{e}=p\left(r-1\right)=3\times \left(3-1\right)=6$$

where, $f_{e}$ is the degree of freedom of the error.

Calculate the degree of freedom of the total fluctuation in Equation (4)

(4) $$f_{T}=pr-1=8$$

where, $f_{T}$ is the degree of freedom of the total fluctuation.

Calculate the variance of the error in Equation (5)

(5) $${\bar{S}}_{e}=S_{e}/f_{e}=0.0031$$

where, ${\bar{S}}_{e}$ is the variance of the error, $S_{e}$ is the sum of the squared fluctuations of the error.

Calculate the power fluctuation and contribution rate in Equation (6)

(6) $${S_{1}}^{\prime }=S_{1}-f_{A}\times {\bar{S}}_{e}=0.15654;\;\rho_{1}={S_{1}}^{\prime }/S_{T}=26.70\%$$

where, ${S_{1}}^{\prime }$ is the fluctuation of power, $\rho_{1}$ is the contribution rate of power.

Calculate the speed fluctuation and contribution rate in Equation (7)

(7) $${S_{2}}^{\prime }=S_{2}-f_{A}\times {\bar{S}}_{e}=0.0788;\;\rho_{2}={S_{1}}^{\prime }/S_{T}=13.43\%$$

where, ${S_{2}}^{\prime }$ is the fluctuation of speed, $\rho_{2}$ is the contribution rate of speed.

Calculate the spacing fluctuation and contribution rate in Equation (8)

(8) $${S_{3}}^{\prime }=S_{3}-f_{A}\times {\bar{S}}_{e}=0.1528;\;\rho_{3}={S_{1}}^{\prime }/S_{T}=26.59\%$$

where, ${S_{3}}^{\prime }$ is the fluctuation of spacing, $\rho_{3}$ is the contribution rate of spacing.

It can be seen from the calculation results each of the parameters have a different effect on the relative density. The laser power has the greatest influence on the relative density of the fabricated part followed by the scan spacing and the scan speed. 

The relation between the various process parameters is given as Equation (9), where, *P* is the laser power in W; *v* is the laser scanning speed in mm/s; *h* is the laser scanning pitch in mm, and *E* is the volume energy density in J/mm^2^ [29].

(9) $$E=\frac{4P}{\pi \cdot v\cdot h}$$

**Figure 4:**
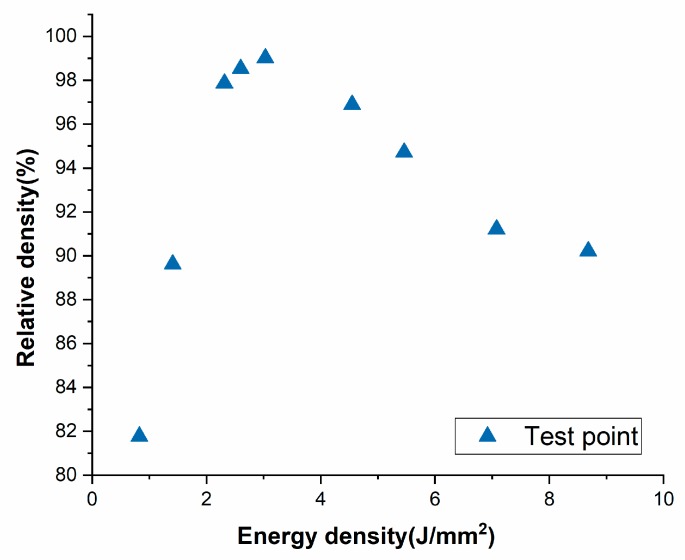
Influence of energy input density on relative density.

The relationship between the laser energy density and the relative density is plotted in Figure 4. The relative density increases first and then decreases with the increase of energy density, and the relative density of components can reach up to 99.01% while the minimum relative density is 81.77%. The reason why the relative density does not reach 100% may be that different defects still exist inside the component, such as holes and cracks. Therefore, research on process parameters and other factors that affect the density of molded parts is very important.

### 2.4. Material Properties Test of 316L Stainless Steel

**Figure 5:**
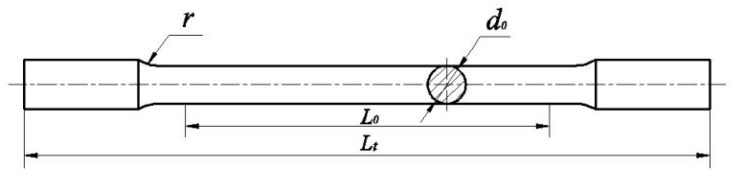
Geometry and dimensions of the standard test specimens.

The standard dog bone tensile specimens were fabricated employing the process parameters (*P* = 150 W, *v* = 700 mm/s, *h* = 0.09 mm) that produced highest relative density. The geometry and dimensions of the standard test specimens is provided in Figure 5, where *d_0_* = 10 mm, *L_0_* = 10 mm, *d_o_* = 100 mm, *L_t_* = 180 mm, transition arc radius *r* = 10 mm ≥ 0.75 *d_0_*. Three specimens were fabricated for conducting the tensile test, and the average values of the mechanical properties were taken as the representative values.

The mechanical testing is conducted using the WDW-600E microcomputer-controlled electronic universal testing machine produced by Shenzhen San-si Vertical and Horizontal Technology Co., Ltd (Shenzhen, China). The tensile test is carried out in accordance with GB/T228.1-2010 at room temperature with a displacement rate 0.5 mm/min. An extensometer is used to measure the longitudinal strain of the specimen and the measurements in the elastic regime are used to evaluate the Young’s modulus. The tensile fracture of the specimen was observed using SEM. The hardness test is carried out using Digi-Rock DR3 Rockwell hardness tester produced by Dongguan Zhong-wang Precision Instrument Co., Ltd. (Dongguan, China). The hardness tests were conducted according to the GBT230.1-2009 metal Rockwell hardness test method.

The standard test piece fabricated by SLM is anisotropic, and defects are likely to occur at the boundary of the molten pool [30,31,32,33,34,35]. The solid solution and aging treatment can eliminate the defects at the boundary of the molten pool, and thus improve the tensile strength. Besides this, due to the increase of austenite after high temperature treatment, a large amount of carbides is dissolved into the austenite, which increases the toughness of the metal [36,37,38]. In this study, the solid solution temperature is 1050 °C and the time is 30 minutes, followed by aging treatment. The aging treatment time is performed for 24 hours at a temperature of 750 °C [36,37,38]. The average values of the mechanical properties are summarized in Table 4. The results show that the tensile strength of 316L stainless steel fabricated by SLM under the optimal process parameters can reach 637.80 MPa, the elongation at fracture can reach 32.10%, and the Rockwell hardness is 68.3 HRB. The tensile strength is 41.73% higher, the elongation at fracture is 19.75% lower, and the Rockwell hardness is 24.1% lower than the values prescribed by ASTM standard for casted specimens.

**Figure 6:**
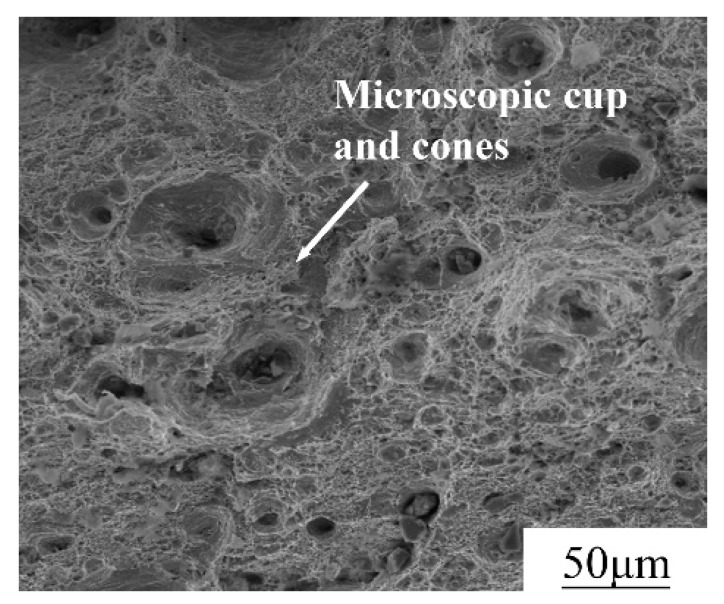
SEM images of the fracture surfaces.

**Figure 7:**
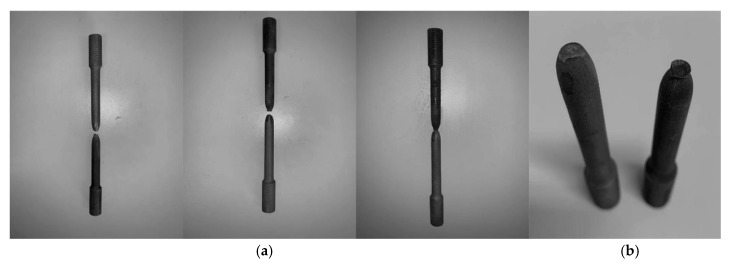
(**a**) Three sets of standard tensile test pieces after breaking; (**b**) Necking phenomenon of tensile test piece.

Figure 6 shows the fracture morphology of the tensile specimens. The elongation of 316L stainless steel at tensile fracture is 32.1%, which indicates that the plasticity of the fabricated parts is suitable for engineering applications. From fracture morphology, it can be found that there are a large number of microscopic cup and cones. Since the microscopic cup and cones are indicative of ductile fracture, 316L stainless steel piece is ductile fracture. During the tensile test, all tensile specimens showed significant necking. As shown in Figure 7, the cross-sectional area of the test piece decreased with the increase of the tensile force.

**Figure 8:**
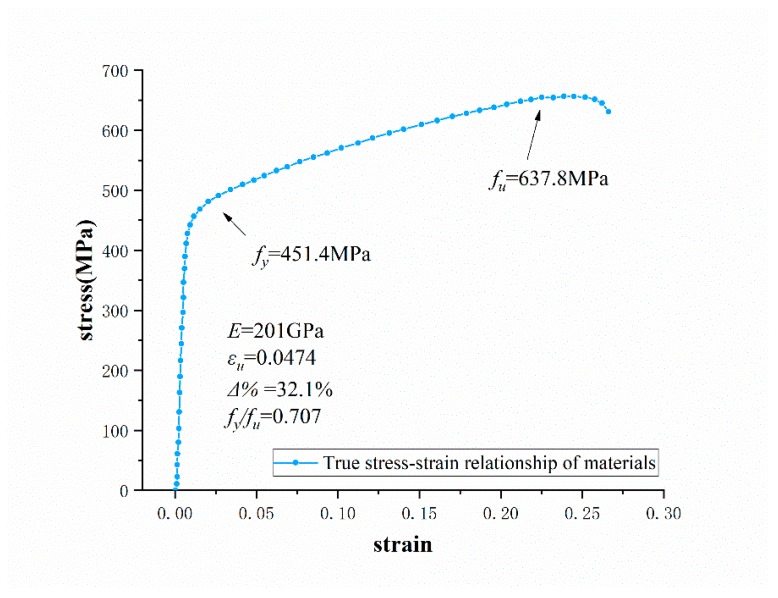
True stress–strain curve based on standard tensile test.

The stress–strain curve of the post-treated standard tensile test piece is shown in Figure 8. *E* is the elastic modulus of the 3D printed stainless steel 316L; *Δ%* is the elongation at fracture; $\varepsilon_{u}$ is the ultimate strain; $f_{y}$ is the yield strength and $f_{u}$ is the ultimate tensile strength. The material constitutive model in the finite element analysis will be determined based on the true stress–strain relationship of the sample.

### 2.5. Modeling Trifurcated Steel Joints

**Figure 9:**
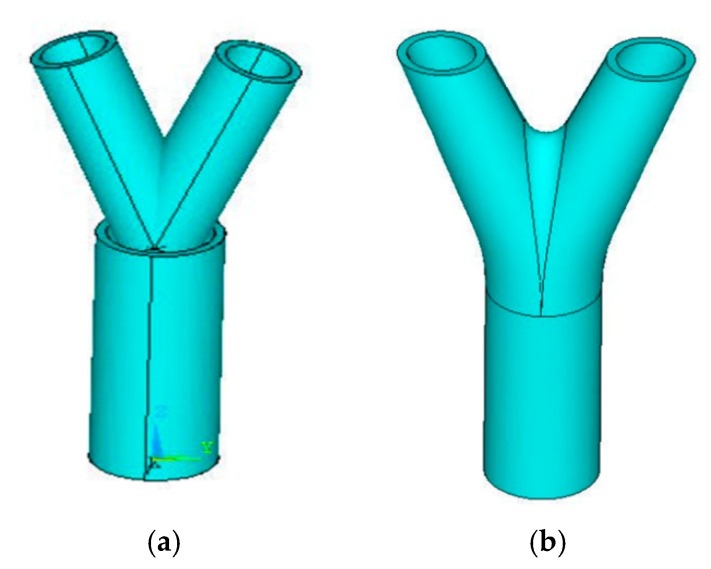
Model creation, (**a**) by ANSYS; (**b**) by SolidWorks.

For the modeling of bifurcation or trifurcation joints, there is chamfer at the intersection. The joint model directly established by finite element software such as ANSYS (ver. 16.0, ANSYS, Inc., Pittsburgh, PA, USA) is shown in Figure 9a, and a smooth transition cannot be achieved between the bottom tube and the branches. In order to solve this problem, this paper used the SolidWorks developed by Dassault to create the model of the trifurcated steel joints. The feature modeling module in SolidWorks can realize smooth transition of joints, as shown in Figure 9b. Compared with the finite element software model system such as ANSYS, SolidWorks has a real parametric feature modeling function in the modeling of complex models, and the tree bifurcation joint model can achieve smooth transition.

### 2.6. Manufacturing Trifurcated Steel Joints Using SLM Process

**Figure 10:**
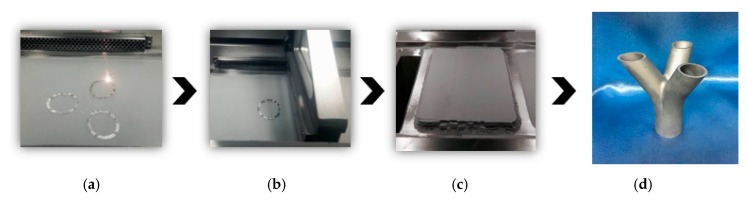
Printing process of SLM technology: (**a**) the laser beam is printed according to the planned path; (**b**) after finishing printing of one layer, put powder on the layer to continue printing; (**c**) the manufacturing of the model is completed after a layer-by-layer scan; (**d**) after cleaning the powder, a trifurcated steel joints model was obtained.

**Figure 11:**
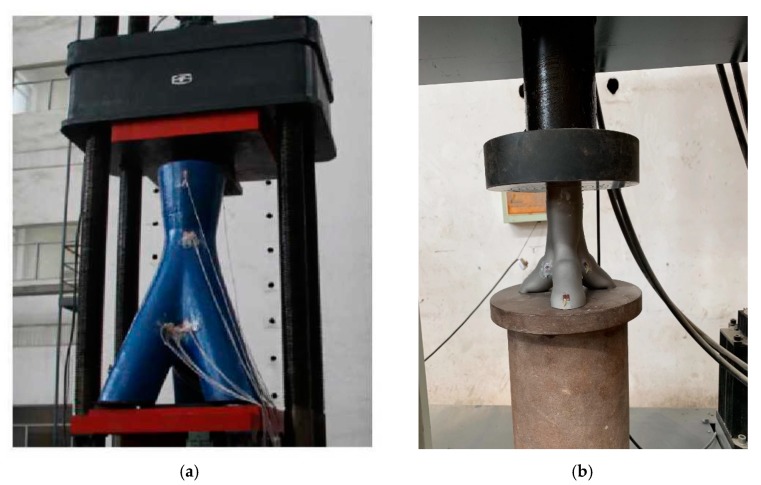
(**a**) Full-scale model of a cast steel bifurcation joints. (**b**) A scale model of the trifurcated steel joints made by SLM.

The joint models created by SolidWorks are converted into STL file format, and then imported into the slicing software of the Additive Manufacturing device for Gcode compilation. The Gcode is a numerical control programming language to guide the CNC nozzle of the additive manufacturing device to perform layer-by-layer printing. It contains the outline and processing path of the entire model. The printing process of the three-fork joint is shown in Figure 10. At first, the additive manufacturing device prints the three branches according to the path planned by the slicing software. Then, it gradually overlays up to the main pipe until a three-dimensional joint is finally fabricated. When the metal powder of the surface is removed after the fabricating table is raised, a three-forked joint scale model is obtained. The scale model and the actual joints we previously cast are made at a ratio of 1:10, as shown in Figure 11.

## 3. Mechanical Testing of the Fabricated Joint

### 3.1. Test Scheme and Measuring Point Arrangement

**Figure 12:**
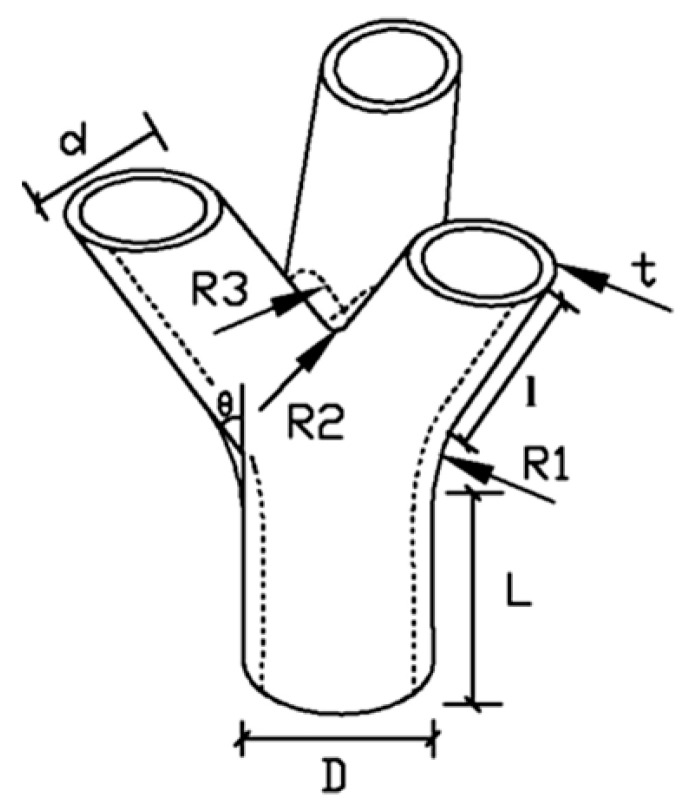
Geometrical features of joint.

The geometric features of the test joint are shown in Figure 12. The size of the joints is designed in accordance with the Chinese structural steel code GB500017-2003. The dimensions of the joint are shown in Table 5. It can be inferred from the table that the dimensional error of the SLM forming node can be controlled within 0.3 mm. The maximum value of relative error is 3.40%. In the X-Y plane, this error is mainly affected by the width of the molten pool, and the width of the molten pool depends on the choice of process parameters. In other words, different process parameters can produce different laser energy densities, and different laser energy densities can produce different melt pool widths. The laser energy density generated by the process parameters selected in this paper is relatively small, resulting in a small weld pool width, which ultimately leads to a slightly smaller measurement size. In the Z-axis direction, it is mainly affected by the cumulative error between layers. Because there is an error between the set layer thickness and the actual layer thickness formed after melting, this error causes the accuracy of the nodes in the Z axis direction to decrease. Overall, the maximum value of relative error is less than 5.00%, which shows all of them satisfy the design requirements.

**Figure 13:**
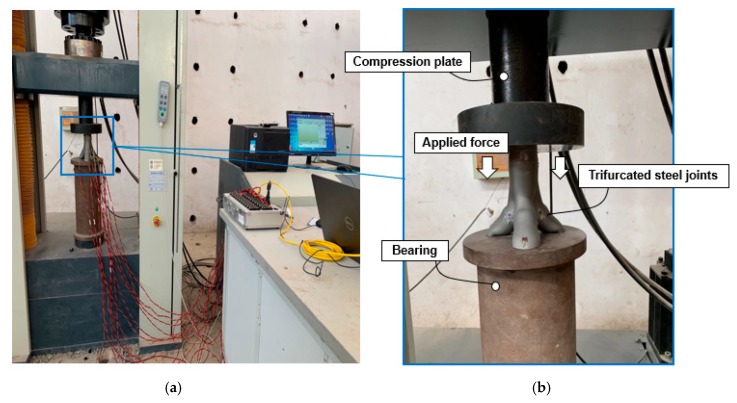
Testing of additive manufactured trifurcated joint: (**a**) Schematic diagram of the experimental setup; (**b**) an enlarged view of the local position.

For bifurcated nodes to be used in a large-span space structures such as the examples provided in Figure 1, the nodes should be capable of carrying large loads. Therefore, the bearing capacity of the joint under axial forces is investigated in this section. The axial compression test was carried out on the fabricated scaled model. The axial compression test was performed employing WDW-600E microcomputer-controlled electronic universal testing machine produced by Shenzhen San-si Vertical and Horizontal Technology Co., Ltd (Shenzhen, China). The surface of the scaled joint is processed in order achieve full contact between the strain gauges and the AM joint. To this end, the residual printing metal powder material on the surface of the joint where the strain gauge is placed is removed. A schematic of the test specimen along with the loading device is shown in Figure 13.

The joint was preloaded to check the contact between the joint and the mechanical test device, to assess the strength and rigidity of the test loading device, and to verify the functionality of the overall test set-up. The maximum load for preloading was 1 kN. After the preloading process was completed, the actual loading was performed after confirming the validity of the preloading data. The actual loading was 5 kN/s, and the test was terminated when the AM joint could not bear any further load.

**Figure 14:**
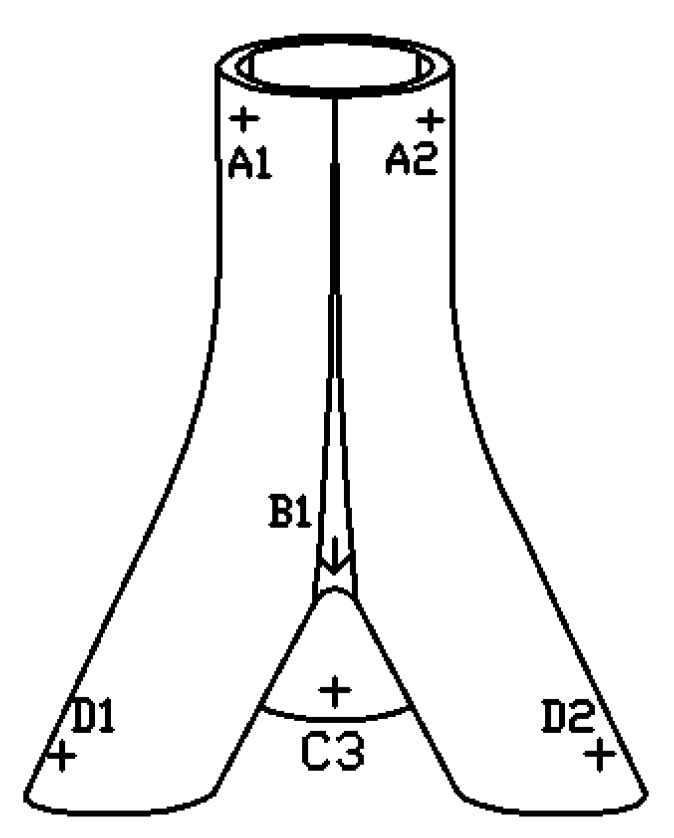
Distribution diagram of measurement points for the joints model.

Resistance strain gauges are employed to collect the strain data. Based on the finite element analysis results of a similar cast steel joint [39], the position of the strain gauges on the 3D printed three-branched joint were arranged in three areas, namely the upper part of the main tube, the vicinity of the joint core area and the end part of the branch pipes. Figure 14 shows the location of the strain measuring points and the number of measuring points is listed in the Table 6. Three strain gauges were uniformly arranged along the circumference considering symmetry. According to the strain measured by the strain gauge, the stress corresponding to each measuring point is calculated to discuss the stress distribution in the 3D printed joint.

### 3.2. Test Results and Analysis

#### 3.2.1. Load–Displacement Curve Analysis

**Figure 15:**
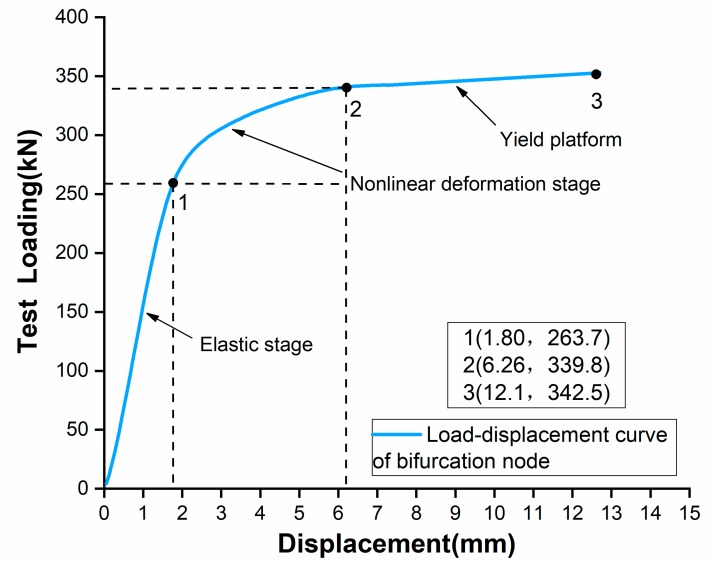
Load–displacement curve of the specimen.

The load-displacement curve of the joint is shown in Figure 15. In the initial stage the load-displacement curve of the specimen increases linearly, indicating that the joint is in the elastic stage. After continuing to increase the load to 263.7kN, the load-displacement curve begins to exhibit a non-linear behavior and the slope of the curve gradually decreases. When the load reaches the peak value of 339.8kN, the curve does not have a sudden decrease in the load carrying capacity, and a yield platform is observed over a large displacement range, indicating that the test piece has good ductility. By comparing the performance of the additive manufactured joint in this study with the results of the reference cast steel three-branched joints under axial compression [40,41], it is found that the joints fabricated by Additive Manufacturing are superior to the traditional cast steel joints in compressive performance and ductility.

The limit load of a joint is usually defined as the load at point 3 in the curve, because the load at this point reaches a maximum value. However, the deformation of the joint also needs to be considered when defining the limit load as excessive deformation of the joint will affect the safety of the overall building. As the joint manufactured by the SLM process is more ductile when the joint reaches point 3, a large deformation is observed when compared to the reference cast joint, and this amount of deformation is not desirable in structural engineering. In addition, based on the experiment we found that when the joint enters the yield platform, the joint deforms rapidly. Therefore, point 2 where the joint enters in to the yielding platform is added to the curve as a control value [42]. Finally, the load carried by the joint at point 3 is compared with the load capacity at point 2 corresponding to the maximum deformation in the safe range, and the smaller value (point 2) is recommended as the limit load of the joint for engineering design purposes [43].

#### 3.2.2. Load–Strain Curve Analysis

**Figure 16:**
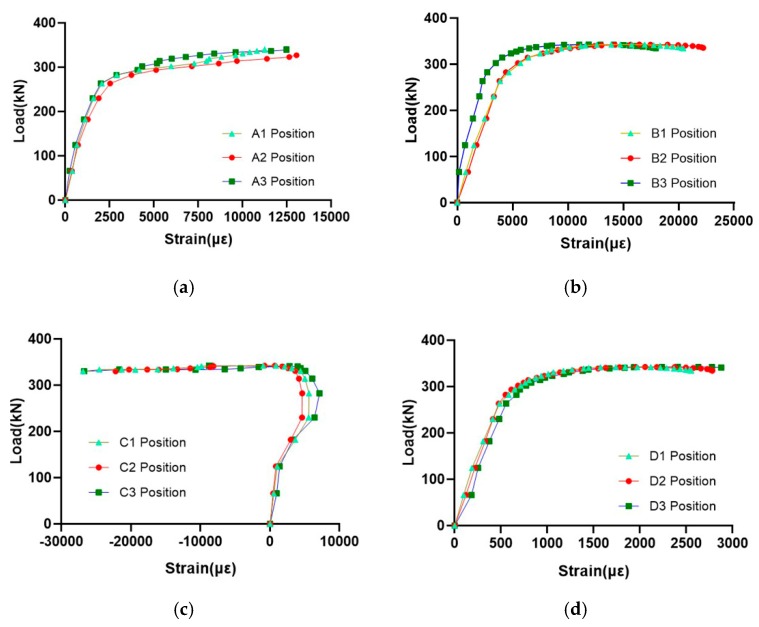
Load–strain curve of different measuring points: (**a**) point a; (**b**) point b; (**c**) point c; (**d**) point d.

After the test, the strain at each measuring point can be obtained from the strain gauge data, and then the corresponding stress value of each measuring point can be obtained. In order to analyze the performance of the AM joint more intuitively, the load-strain curve at all the measurement points is drawn, as shown in Figure 16.

In the initial stage of deformation, the strain of each measuring point increases linearly with the increase of the load. When the load reaches the maximum, the strain value inside the branch end of the AM node is the largest, followed by the strain measured in the vicinity of the joint core. This indicates that the plastic zone first appears on the inside of the branch end of the node, and as the load is continuously increased, the vicinity of the joint core area also enters the plastic phase.

By comparison, the strain values on the upper part of the main pipe and outside the end of the branch are relatively small, and the strain values at the inner side of the branch end and the vicinity of the joint core area are relatively large. This indicates that the stresses of the inner side of the branch end and the vicinity of the joint core area are relatively large, which is where the stress is unfavorable, and the stress distribution in other parts is relatively small. Therefore, the load-strain data of the test indicates that the inner side of the branch end and the vicinity of the joint core area of the printed node are the most unfavorable positions, and the node is prone to damage in this area.

The measuring points are arranged symmetrically along the circumference of the joint. The stress levels of these symmetrically distributed measuring points are basically the same, indicating that the load is substantially along the axial direction and the eccentricity is small.

## 4. Numerical Simulation of 3D Printed Joint

### 4.1. Finite Element Analysis Model

The SolidWorks software is used to model the nodes to ensure that the established node model is consistent with the actual fabricated nodes, and has a smooth transition shape, which is then imported into ANSYS for finite element analysis. An Elastoplastic stress-strain relationship with bilinear hardening and a von Mises yield criterion are used in this paper. The parameters of the constitutive model were obtained by fitting the experimental data.

### 4.2. Destruction Process and Plastic Zone Expansion

**Figure 17:**
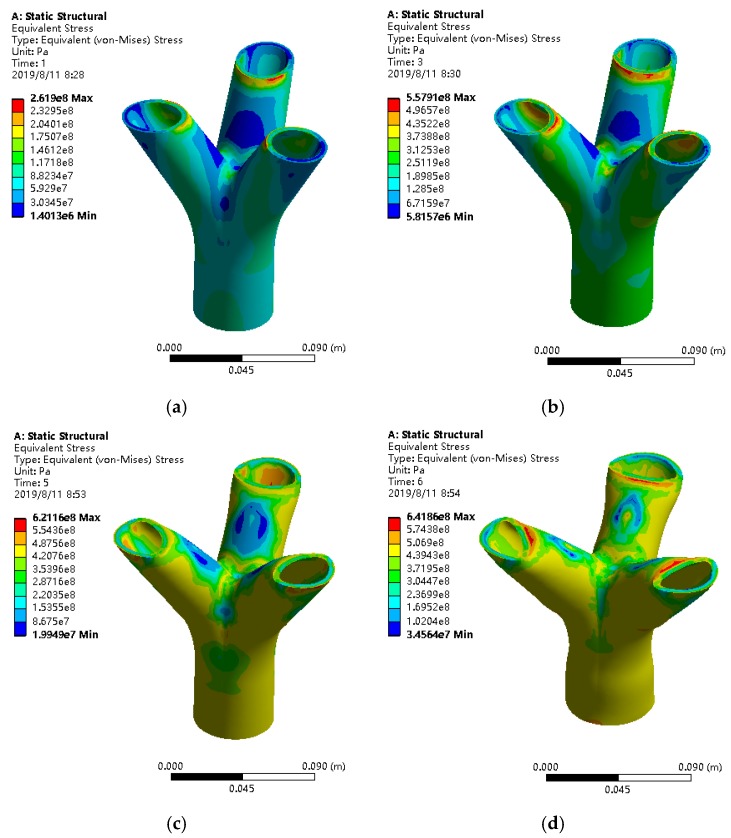
Stress cloud diagram of joints under different loads: (**a**) When the load is 100 kN; (**b**) When the load is 200 kN; (**c**) When the load is 300 kN; (**d**) When the load reaches the maximum load.

Figure 17 shows the stress contour diagram of the joints when the load reaches 100 kN, 200 kN, 300 kN and maximum load in a sequence. It can be seen from the figure that the overall stress level of the joint under the load of 100 kN is low, the minimum stress value is mainly distributed outside the end of the branch pipe, and the maximum stress value is mainly distributed inside the end of the branch pipe. When the load reaches 200 kN, part of the joint enters the plastic state, and the stress levels in the joint gradually increase with the increase of the load. A small amount of yielding occurs on the inside and the end part of the branch. As the load gradually increases, the yielding area gradually expands. When the load reaches 300 kN, the plastic zone begins to appear in the vicinity of the joint core area. After the load reaches the maximum value, the stress values on the inner side of the branch end and the vicinity of the joint core area are both relatively large, and obvious plastic deformation occurs, which eventually leads to joint failure. In summary, the stress distribution of bifurcated joints under compression exhibits local variations. The yielding phenomenon first appeared on the inner side of the end of the joint. With the continuous increase of the load, the accumulation of increase in stress in the vicinity of the joint core area gradually appeared, and the stress in other parts was small. The inner side of the branch end of the joint and the vicinity of the joint core area have a greater impact on the ultimate bearing capacity of the joint.

### 4.3. Comparative Analysis with Test Results

**Figure 18:**
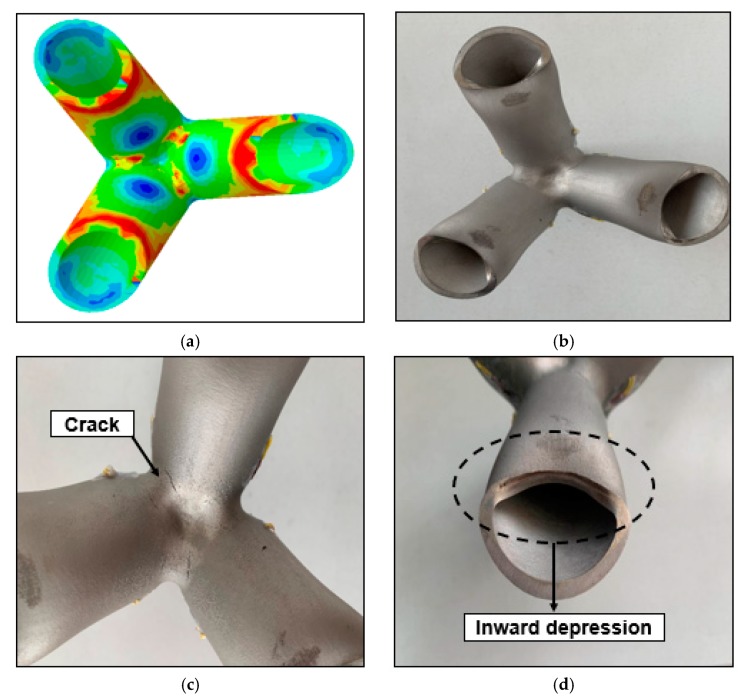
Results comparison: (**a**) numerical result; (**b**) test result; (**c**) end detail; (**d**) intersection detail.

Figure 18 is a comparison of ultimate deformation mode between the test result and the numerical result. Both the test and the numerical results indicate that there is a large stress in the core region of the main branch intersection and inside of the branch end, as shown in Figure 18a,b. Due to the thick wall of the joint pipe and the good plasticity of the material, the failure of the joint is often due to the rapid expansion of the plastic zone with the increase of the load after entering the yielding stage, which eventually causes the joint to deform excessively and cannot continue to be stressed. In this test, when the joint enters the plastic phase, a significant local buckling and concave deformation occurs at the end of the pipe branch due to compression, as shown in Figure 18c. When the load frame continues to apply load for a period of time, cracks begin to appear in the core area where the main pipe and the pipe are connected, as shown in Figure 18d. Continued loading at this time will cause the cracks to develop in a direction perpendicular to the branch, eventually cause the joint to undergo strength damage.

**Figure 19:**
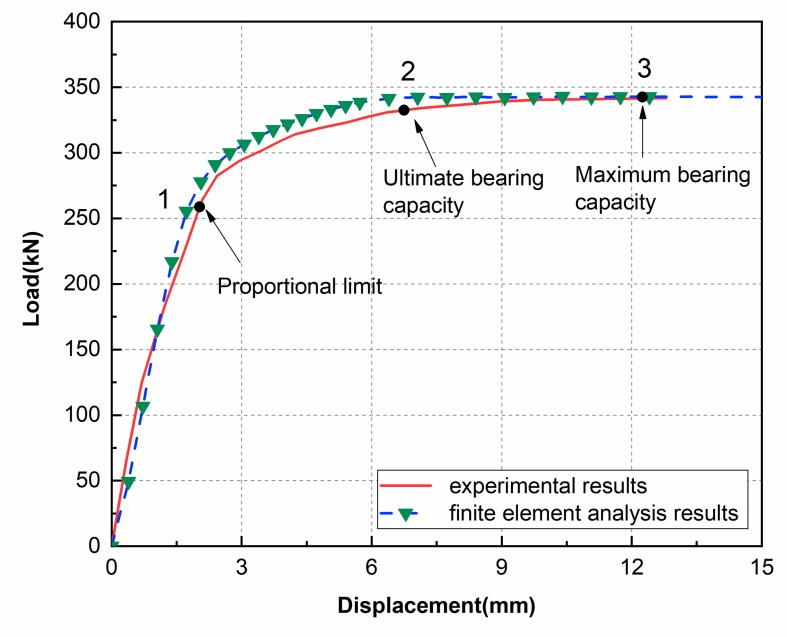
Comparison of load–displacement curves between FEM and test results.

To further verify the accuracy of the finite element model, Figure 19 shows the comparison of the load–displacement curve obtained from the finite element simulation with the experimental results. It can be seen that they are basically the same, and the ultimate deformation mode of the finite element model is consistent with the deformation mode of the experiment. From the three feature points, the average difference is only −1.59% and the standard deviation is 7.76%. The finite element model is accurate and can be used to predict the performance of tree-like bifurcation nodes for AM under axial compression.

## 5. Conclusions

This body of work demonstrates the use of AM technology to manufacture trifurcated steel joints and characterizes the mechanical properties of AM joints. The following conclusions can be obtained from this study.

(1) The quality of the 3D printed parts is significantly affected by the process parameters. Nine orthogonal tests show that the preferred process parameters for SLM fabricated stainless steel 316L are laser power *P* = 150 W, scanning speed *v* = 700 mm/s, scanning pitch *h* = 0.09 mm, print layer thickness 30 µm.

(2) Mechanical tests on test specimens show 3D printed members have good mechanical performance and high geometrical precision. Experimental studies on scaled joints indicate that the AM joints exhibit similar plastic zone, load-displacement behavior and ultimate deformation mode as that of the casted joints. Furthermore, the numerical simulations AM joint agree well with the experimental results.

(3) The additive manufacturing technology is proved to be a convenient method for the production of complex spatial structural nodes and can be used to fabricate other new building components which can provide designers with wider design options.

## Figures and Tables

**Table 1:** Factors and levels for orthogonal test

Level	Laser Power (W)	Scanning Speed (mm/s)	Scanning Pitch (mm)
1	50	200	0.07
2	100	450	0.09
3	150	700	0.11

**Table 2:** Experimental design and results of specimen

Test Number	A (P)	B (V)	C (h)	Relative Density (%)	*SNR* (dB)
1	50	200	0.07	96.89	−0.137
2	50	450	0.09	89.62	−0.476
3	50	700	0.11	81.77	−0.874
4	100	200	0.09	91.21	−0.400
5	100	450	0.11	97.86	−0.094
6	100	700	0.07	98.53	−0.064
7	150	200	0.11	90.21	−0.447
8	150	450	0.07	94.71	−0.236
9	150	700	0.09	99.01	−0.043

**Table 3:** ANOVA calculation table

Category	A (P)	B (V)	C (h)	*SNR* (dB)
$T_{1}$	−1.487	−0.984	−0.437	
$T_{2}$	−0.558	−0.806	−0.919	
$T_{3}$	−0.726	−0.981	−1.415	
${\bar{T}}_{1}$	−0.496	−0.328	−0.146	
${\bar{T}}_{2}$	−0.186	−0.269	−0.306	
${\bar{T}}_{3}$	−0.242	−0.327	−0.472	T=−2.771
Range (R)	0.073	0.031	0.062	
Fluctuation (S)	0.163	0.085	0.159	$S_{T}=0.586$

**Table 4:** Mechanical properties test results

Standard Tensile Specimen	Yield Strength (MPa)	Tensile Strength (MPa)	Elongation at Fracture (%)	Hardness (HRB)
SLM fabrication	451.4 ± 5.6	637.8 ± 9.2	32.1 ± 1.5	68.3 ± 2.9
Forged (ASTM A473)	170	450	40	90

**Table 5:** Actual print size of the test piece

Parts of the Joint	Measured Sizes	Design Sizes	Relative Errors (%)
Main pipe length (L/mm)	79.80	80	0.25
Main pipe diameter (D/mm)	49.92	50	0.16
Main pipe wall thickness (T/mm)	3.98	4	0.5
Branch pipes length (l/mm)	119.79	120	0.17
Branch pipes diameter (d/mm)	34.93	35	0.2
Branch pipes wall thickness (t/mm)	3.45	3.5	1.42
Chamfer (R1/mm)	99.95	100	0.05
Chamfer (R2/mm)	1.94	2	3.00
Chamfer (R3/mm)	4.83	5	3.40

**Table 6:** Locations and notation of measurement points

Position	Strain Gauge		
Upper part of the main pipe	A1	A2	A3
Vicinity of the joint core area	B1	B2	B3
Inside the end part of the branch	C1	C2	C3
Outside the end part of the branch	D1	D2	D3
